# Comprehensive Bioinformatic Characterization of CD70, CD80, and TIGIT as Diagnostic, Prognostic, and Immune Biomarkers in Pan-Cancer

**DOI:** 10.3390/cimb48060641

**Published:** 2026-06-21

**Authors:** Christos Panagiotis Rigopoulos, Ilias Georgakopoulos-Soares, Apostolos Zaravinos

**Affiliations:** 1 Department of Life Sciences, School of Sciences, European University Cyprus, P.O. Box 22006, 1516 Nicosia, Cyprus; cr211316@students.euc.ac.cy; 2 Cancer Genetics, Genomics and Systems Biology Laboratory, Basic and Translational Cancer Research Center (BTCRC), 1516 Nicosia, Cyprus; 3 Division of Pharmacology and Toxicology, Dell Paediatric Research Institute, College of Pharmacy, The University of Texas at Austin, Austin, TX 78712, USA; ilias@austin.utexas.edu

**Keywords:** immunotherapy, multi-omics, pan-cancer, CD70, CD80, TIGIT, immune checkpoints

## Abstract

Immunotherapy has transformed cancer treatment; however, clinical benefit remains limited to a subset of patients, underscoring the need for robust biomarkers that capture tumor-immune interactions across cancer types. In this study, we performed a comprehensive pan-cancer, multi-omics characterization of the immune checkpoint–related molecules CD70, CD80, and TIGIT to evaluate their diagnostic, prognostic, and immunological relevance. Using integrative analyses of transcriptomic, epigenomic, genomic, pharmacogenomic, and single-cell RNA-sequencing data from The Cancer Genome Atlas and complementary resources, we assessed expression patterns, DNA methylation, somatic mutations, copy number alterations, immune infiltration, tumor stemness, and drug sensitivity. CD70, CD80, and TIGIT were broadly dysregulated across multiple malignancies, with coordinated overexpression particularly evident in kidney renal clear-cell carcinoma. Elevated expression of these immune checkpoints was associated with advanced tumor stage, aggressive molecular subtypes, and unfavorable survival outcomes in selected cancers, including uveal melanoma and renal malignancies. Functional analyses revealed significant associations between checkpoint expression and key oncogenic pathways, including epithelial–mesenchymal transition, apoptosis, and hormone receptor signaling, suggesting links with tumor progression and immune activation states. Immune deconvolution analyses indicated that TIGIT expression is associated with a T-cell–inflamed microenvironment and reduced neutrophil infiltration, while CD80 exhibited methylation-dependent associations with immune cell composition. Genomic and epigenetic alterations were found to correlate with checkpoint expression patterns and immune phenotypes across tumor types. Pharmacogenomic profiling identified associations between checkpoint expression and sensitivity to multiple anticancer agents; however, these findings are based on cell line datasets and should be considered predictive. Single-cell transcriptomic analyses further resolved cell-type–specific expression patterns, distinguishing tumor-intrinsic from immune-restricted expression profiles. Collectively, our findings establish CD70, CD80, and TIGIT as integrative biomarkers of tumor progression, immune contexture, and therapeutic response, providing a rationale for their clinical exploitation in precision immuno-oncology.

## 1. Introduction

Cancer remains among the leading causes of morbidity and mortality worldwide [1]. It is a multifactorial disease influenced by both genetic and environmental factors. Germline and somatic mutations are the key genetic contributors [2], while epigenetic changes are also critical regulators [3].

Among the most promising therapeutic strategies is immunotherapy, which harnesses the immune system to recognize and eliminate tumor cells [4]. Despite major advances in this field, the clinical efficacy of immunotherapy remains limited in certain cancer types, largely due to tumor heterogeneity, the complexity of the tumor microenvironment (TME), and immunosuppression. Various immunotherapeutic approaches aim to either stimulate an immune response (active immunotherapy) or reinforce existing responses (passive immunotherapy) [5]. Central to many of these strategies is the modulation of T cell activity, which is regulated through costimulatory and inhibitory molecules expressed at the immunological synapse [6].

While CD70, CD80, and TIGIT are often studied individually, they represent complementary components of the immunological synapse that regulate distinct but interconnected stages of T-cell responses. CD70 primarily promotes T-cell activation and survival through CD27 signalling, CD80 functions as a bidirectional regulator balancing co-stimulatory (CD28-mediated) and inhibitory (CTLA-4-mediated) signals, and TIGIT acts as a key inhibitory receptor associated with T-cell exhaustion. The coordinated dysregulation of these molecules may therefore reflect integrated states of immune activation, suppression, and adaptation within the tumor microenvironment. Investigating them as a combined axis enables a more comprehensive understanding of immune regulation in cancer beyond single-checkpoint analyses.

Among various immune-related molecules, CD70, CD80 and TIGIT have emerged as key players with potential diagnostic, prognostic, and therapeutic value. CD70, a member of the TNF ligand family, is expressed in activated immune cells and mediates T cell costimulation via interaction with CD27 [7]. CD80 (B7-1) belongs to the immunoglobulin superfamily and is involved in T cell activation through CD28 binding but also contributes to immune suppression via interaction with CTLA-4 [8,9]. TIGIT, a PVR-like inhibitory receptor, is widely expressed on T cells and NK cells and binds to CD155, contributing to T cell exhaustion and tumor immune evasion [10,11].

Despite the known individual roles of these molecules, little is known about their combined expression, functional interaction, and prognostic relevance. Here we performed a comprehensive bioinformatic analysis of CD70, CD80 and TIGIT to investigate their expression, their prognostic potential, and immunological roles, as well as their interactions and underlying molecular mechanisms, in pan-cancer.

## 2. Materials and Methods

### 2.1. Data Collection and Processing

We used the Gene Set Cancer Analysis (GSCA) platform, through which we extracted and analyzed the cancer gene set associated with mRNA expression, as well as mutations, immune infiltration, and resistance to various drugs. The mRNA expression, copy number variation (CNV), and methylation data were obtained from UCSC Xena (https://xena.ucsc.edu/, accessed on 14 November 2025) [12]. In addition, single nucleotide variation (SNV) data were collected from the Synapse project (syn7824247). Finally, drug sensitivity information was retrieved from two different databases, GDSC [13,14,15] and CTRP [4,16,17]. Gene expression values from TCGA (tumor samples) and GTEx (normal tissues) were obtained from UCSC Xena as RSEM-normalized, log2-transformed values. Batch effects between TCGA and GTEx datasets were corrected using the ComBat method as implemented in the GSCA and GEPIA2 pipelines prior to differential expression analysis [18,19]. We also used the TISCH2 resource to perform in-depth analyses of our genes via single-cell RNA sequencing [20,21]. The TISCH2 database was used to characterize the TME through cell-by-cell analysis. The TME comprises cancer cells surrounded by diverse non-cancerous cells within a vascularized extracellular matrix, including a wide variety of immune cells, CAFs, endothelial cells (ECs), and pericytes [22]. Patient datasets include cases with no prior therapy as well as those treated with chemotherapy, immunotherapy, or targeted therapy. A uniform analysis pipeline was used for quality control, clustering, and cell-type annotation [20,21].

### 2.2. Differential Expression of CD70, CD80 and TIGIT

We examined differential expression of the three genes across 14 cancer types: bladder cancer, BLCA (n = 19); breast cancer, BRCA (n = 114); colon adenocarcinoma, COAD (n = 26); esophageal carcinoma, ESCA (n = 11); head and neck squamous cell carcinoma, HNSC (n = 43); kidney chromophobe, KICH (n = 25); kidney renal clear-cell carcinoma, KIRC (n = 72); kidney renal papillary cell carcinoma, KIRP (n = 32); liver hepatocellular carcinoma, LIHC (n = 50); lung adenocarcinoma, LUAD (n = 58); lung squamous cell carcinoma, LUSC (n = 51); prostate adenocarcinoma, PRAD (n = 52); stomach adenocarcinoma, STAD (n = 32); and thyroid carcinoma, THCA (n = 59). The *p*-value was estimated using the *t*-test and subsequently adjusted by FDR. A *p* < 0.05 was considered statistically significant.

We categorized samples into low- and high-expression groups and evaluated patient survival (OS, PFS, DFI, and DSS). Cox proportional hazards models and the log-rank test were used for analysis, and results were presented with Kaplan–Meier curves.

To explore differences in gene expression, we used GEPIA2, which analyzes TCGA and GTEx data through a standard pipeline [23]. Additionally, we examined expression changes among cancer subtypes using Wilcoxon and ANOVA tests. We also analyzed the association between gene expression and disease stages using data from 9478 samples across 27 cancer types, as previously analyzed [24]. Stages were categorized as I, II, III, and IV. In contrast, IGCCCG classification grouped samples into good, intermediate, and poor. Trend analysis was performed using the Mann–Kendall test.

We further quantified pathway activity levels between high- and low-expression groups using pathway activity scores (PAS). RPPA (Reverse Phase Protein Assay) data were collected from TCPA via GSCA [23]. These scores reflect protein-level pathway activity and are distinct from transcriptomic enrichment methods such as GSVA or ssGSEA. Differences in PAS between high- and low-expression groups were assessed using *t*-tests, with *p*-values adjusted by FDR (Benjamini–Hochberg method). FDR < 0.05 was considered statistically significant. Group differences were assessed with *t*-tests. Multiple testing correction was performed using the Benjamini–Hochberg false discovery rate (FDR) method. Unless otherwise specified, statistical significance was defined as FDR-adjusted *p* < 0.05.

### 2.3. Immune Infiltration and Expression Patterns of CD70, CD80, TIGIT in Immune Cells

For immune-cell analyses, we evaluated 4950 samples from 33 cancer types. The abundance of 24 immune-cell types was estimated using ImmuCellAI (http://bioinfo.life.hust.edu.cn/ImmuCellAI/#!/, accessed on 16 November 2025). We then correlated gene expression with immune-cell scores using Spearman’s test, setting statistical significance at 0.05 [25]). ImmuCellAI infers cell-type proportions from a curated 788-immune-gene signature encompassing lineage-defining markers for 24 immune cell types. CD70, CD80, and TIGIT are not among the defining marker genes for the immune cell populations most strongly correlated with their expression (e.g., CD8^+^ T cells, exhausted T cells, neutrophils), minimizing potential circularity in the correlation analyses. Nonetheless, all immune infiltration results are interpreted as correlative associations and not as direct measures of immune cell abundance. Multiple testing correction was performed using the Benjamini–Hochberg false discovery rate (FDR) method. Unless otherwise specified, statistical significance was defined as FDR-adjusted *p* < 0.05.

### 2.4. Immune Infiltration and Genetic Alterations (CNVs, SNVs)

We assessed differences in immune-cell expression between wild-type (WT) and mutated (SNV) groups using the Wilcoxon test. We then examined correlations between SNVs/CNVs affecting our genes and immune-cell populations using Spearman’s test.

### 2.5. Immune Infiltration and Methylation

Within the immune-cell framework, we also evaluated correlations with an epigenetic modification—DNA methylation—again using Spearman’s test.

### 2.6. Genetic Mutations

We investigated SNVs across 10,234 samples from 33 cancer types [18,19]. We focused on protein-altering mutations, such as missense and nonsense variants, and frame-shifting insertions/deletions. We also assessed survival differences between mutation-positive groups and WT groups. CNV data were obtained from 11,495 TCGA samples, and significantly deleted or amplified regions were analyzed with GISTIC 2.0. Samples were categorized as WT, Amp, and Del. Survival differences between groups were computed using log-rank tests and the R survival package (v.3.8-6).

### 2.7. Differential Methylation

We examined differential methylation between normal and tumor samples using Illumina Human Methylation 450 K data. Spearman’s test was additionally used to identify correlations between gene-expression patterns and methylation levels. Samples were split into high- and low-methylation groups based on the mean methylation level. Survival differences between groups were evaluated with the R survival package.

### 2.8. Correlation Between Genes and Drug Sensitivity

We collected half-maximal inhibitory concentrations (IC50) for a broad range of drugs tested across numerous cell lines, along with corresponding gene-expression profiles, from the Genomics of Drug Sensitivity in Cancer (GDSC) resource [13,14,15]. We correlated gene expression with drug IC50 values using Pearson’s test and adjusted *p*-values by FDR. The same procedure was performed using data from the Cancer Therapeutics Response Portal (CTRP) [17,26].

### 2.9. Pan-Cancer Stemness Correlation Analysis

Pan-cancer analyses were performed using data from The Cancer Genome Atlas (TCGA). Tumor stemness was evaluated using two previously established indices: the DNA methylation–based stemness score (DNAss) and the RNA expression–based stemness score (RNAss), which quantify the degree of similarity between tumor samples and stem cell–like epigenetic or transcriptional profiles, respectively.

Normalized RNA-sequencing expression data for CD70, CD80, and TIGIT were extracted for all available TCGA tumor types. Gene expression values were transformed using a log2(x + 0.001) transformation prior to analysis. Pearson’s correlation analysis was used to assess the association between gene expression levels and stemness scores (DNAss or RNAss) within each tumor type. Correlation coefficients (R) and corresponding *p*-values were calculated using two-sided Pearson correlation tests. Correlations were considered biologically relevant when |R| > 0.3 and statistically significant after FDR correction.

## 3. Results

### 3.1. Expression Profiling of CD70, CD80, TIGIT Molecules in Pan-Cancer

**Figure 1:**
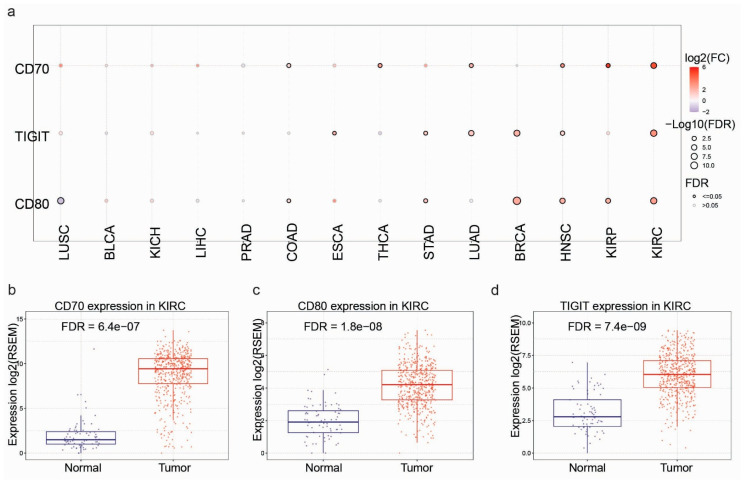
Differential expression profiling of CD70, CD80, and TIGIT across human cancers. (**a**) Differential mRNA expression (log_2_fold change) of CD70, CD80, and TIGIT, integrating data from TCGA and GTEx databases. Red indicates over-expression; blue indicates down-expression in tumor versus normal tissues. (**b**–**d**) Box plots depicting mRNA expression (log_2_RSEM) of CD70 (**b**), CD80 (**c**), and TIGIT (**d**) in kidney renal clear-cell carcinoma (KIRC) against matched normal kidney tissues.

To evaluate the potential of CD70, CD80, and TIGIT as diagnostic biomarkers, we analysed their mRNA expression across multiple cancer types using integrated data from The Cancer Genome Atlas (TCGA) and the Genotype-Tissue Expression (GTEx) database. Pan-cancer analysis revealed widespread dysregulation of these genes across diverse malignancies, predominantly characterised by upregulation—particularly for CD80 and TIGIT—in several solid tumors, including kidney renal cell carcinoma (KIRC), breast invasive carcinoma (BRCA), and lung squamous cell carcinoma (LUSC) (Figure 1a). Among all tumor types, KIRC exhibited the most pronounced upregulation (Figure 1b–d). Importantly, independent validation in additional high-prevalence cancers confirmed significant CD80 overexpression. For example, CD80 expression was markedly elevated in BRCA (FDR = 3.6 × 10^−13^) (Figure S1a) and LUSC (FDR = 7.1 × 10^−9^) (Figure S1b), supporting its relevance across multiple tumor contexts.

### 3.2. Association of CD70, CD80, and TIGIT Expression with Clinicopathological Features and Molecular Subtypes

**Figure 2:**
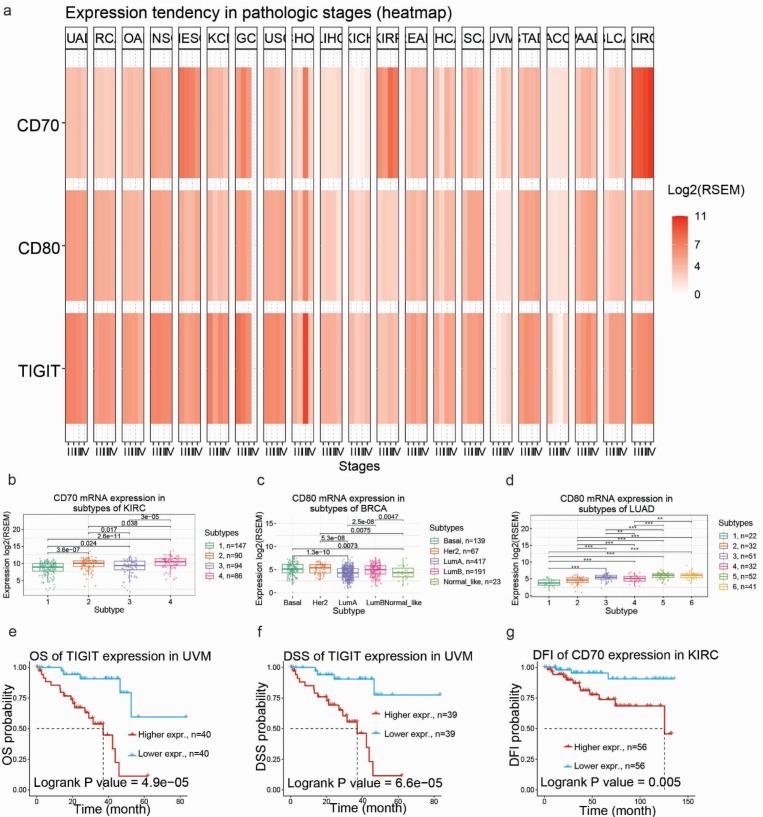
Clinical and prognostic relevance of CD70, CD80, TIGIT. (**a**) Heatmap illustrating the correlation between gene expression and pathological tumor stage across multiple cancer types. Color intensity represents the degree of expression change with advancing disease stage. (**b**) Molecular subtype-specific expression of CD70 in KIRC. (**c**) Molecular subtype-specific expression of CD80 in breast invasive carcinoma (BRCA). (**d**) CD80 expression across molecular subtypes of lung adenocarcinoma (LUAD). **, *p* < 0.01; ***, *p* < 0.001. (**e**–**f**) Kaplan–Meier survival analysis for TIGIT in UVM, showing OS (**e**) and DSS (**f**). Patients were stratified by median TIGIT expression into high- and low-expression groups. (**g**) Disease-Free Interval (DFI) analysis for CD70 in KIRC. Statistical significance was determined by log-rank test. HR, hazard ratio; CI, confidence interval.

To evaluate the clinical trajectory of CD70, CD80, and TIGIT, we investigated their correlation with pathological progression and distinct molecular landscapes. Global stage-dependent profiling indicated that immune checkpoint dysregulation is not merely a static feature of malignancy but appears to be associated with advancing disease states, especially in KIRC and ACC (Figure 2a). This phenomenon was most clearly exemplified in KIRC, where we observed a striking, stepwise escalation of CD70 mRNA levels tracking with tumor aggressiveness. Expression was significantly higher in stage III compared to early-stage disease and reached the highest levels in stage IV (Figure 2a). This progressive upregulation was paralleled by CD80, which similarly displayed peak expression in stage IV renal tumors (Figure S2a), and was further corroborated in thyroid carcinoma (THCA), where late-stage tumors exhibited significantly higher CD70 (Figure S2b). Beyond physical staging, molecular stratification of KIRC and BRCA revealed profound heterogeneity, with higher CD80 levels in metabolic (subtype 2) and distinct (subtype 4) KIRC tumors (Figure 2b), as well as in the aggressive Basal-like subtype of breast tumors (Figure 2c). In the case of breast tumors, the basal-like subtype clinically overlaps with triple-negative BRCA and shows significantly higher expression compared to luminal A and luminal B phenotypes. Similar subtype-specific variation was evident in lung adenocarcinoma (LUAD) (Figure 2d), underscoring the potential for using these markers to stratify patients not only by cancer type but also by specific molecular vulnerabilities.

### 3.3. Prognostic Significance of the CD70-CD80-TIGIT Axis in Predicting Survival Outcomes

Having established the dysregulation and stage-associated patterns of these checkpoints, we next evaluated their prognostic relevance.

In uveal melanoma (UVM) and thymoma (THYM), elevated TIGIT expression was significantly associated with poorer clinical outcomes. Kaplan–Meier analysis demonstrated that patients with high TIGIT expression had reduced overall survival (OS) compared with those with low expression (log-rank *p* = 4.9 × 10^−5^) (Figure 2e and Figure S3). This association was consistent in disease-specific survival (DSS) analysis (log-rank *p* = 6.6 × 10^−5^) (Figure 2f).

Similarly, high CD70 expression was associated with shorter disease-free interval (DFI) in KIRC (log-rank *p* = 0.005) (Figure 2g), suggesting potential clinical relevance in recurrence risk stratification.

However, these findings should be interpreted with caution. Due to incomplete clinical annotation across TCGA cohorts, multivariate analyses adjusting for confounding variables (e.g., stage, treatment, and comorbidities) were not feasible. Therefore, the reported associations are correlative and hypothesis-generating, rather than indicative of independent prognostic effects.

### 3.4. Functional Enrichment Analysis and Correlation with Canonical Oncogenic Signaling Pathways

**Figure 3:**
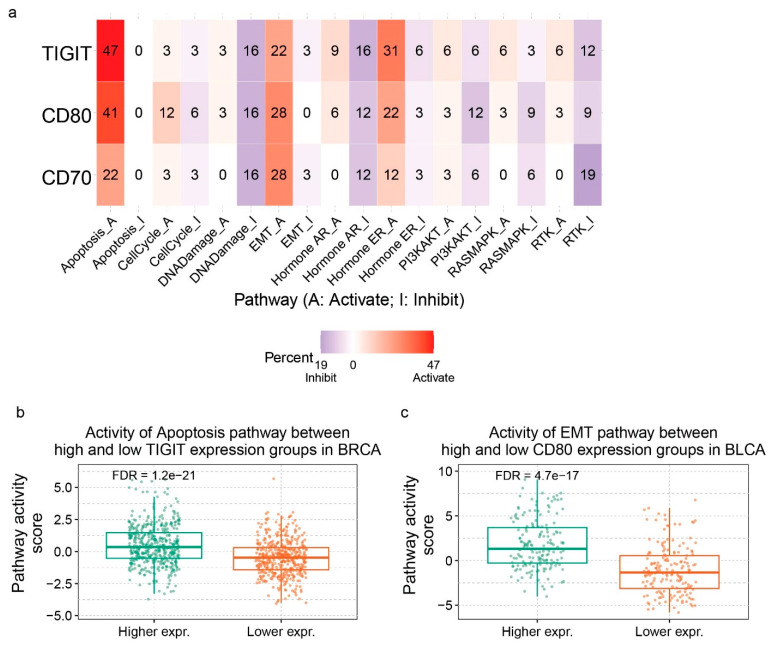
Functional enrichment analysis and pathway correlations. (**a**) Heatmap depicting the correlation between CD70, CD80, and TIGIT expression and canonical oncogenic pathway activity scores across pan-cancer cohorts. Red indicates positive correlation (pathway activation); blue indicates negative correlation (pathway inhibition). (**b**) Scatter plot showing the positive correlation between TIGIT expression and the apoptosis pathway activity score in BRCA. (**c**) Scatter plot demonstrating the positive correlation between CD80 expression and the epithelial–mesenchymal transition (EMT) pathway score in bladder urothelial carcinoma (BLCA).

To elucidate the molecular mechanisms underlying the observed prognostic associations, we analyzed the correlation between CD70, CD80 and TIGIT expression and the activity of canonical cancer pathways. A global functional landscape analysis suggests that this immune checkpoint expression is not only associated with T-cell exhaustion but also correlates with core oncogenic signaling pathways, specifically those regulating cell survival and invasion (Figure 3a). The summary heatmap highlights a consistent pattern in which higher expression of TIGIT and CD80 is associated with activation of epithelial–mesenchymal transition (EMT) pathways and inverse association with programmed cell death (apoptosis) across multiple tumor types.

In BRCA, where we previously identified elevated TIGIT expression, functional analysis uncovered a significant positive correlation between TIGIT levels and apoptotic signaling. Contrary to the immune-suppressive role typically ascribed to checkpoints, high TIGIT expression was strongly associated with increased apoptosis pathway activity (FDR = 1.2 × 10^−21^) (Figure 3b). This may reflect a “hot” tumor microenvironment characterized by high immune infiltration and ongoing immune-mediated tumor cell killing. This apparent paradox—an inhibitory checkpoint correlating with apoptosis activation—is consistent with previous biological observations in the context of triple-negative breast cancer, which is characterized by dense T-cell infiltration and an inflammatory microenvironment. In this setting, TIGIT upregulation likely represents a reactive, feedback inhibitory response to intense immune activation rather than primary immunosuppression.

Parallel analysis in bladder urothelial carcinoma (BLCA) identified CD80 as a gene whose expression was significantly associated with features linked to metastatic progression. We observed a highly significant positive correlation between CD80 expression and the activation of the EMT pathway (FDR = 4.7 × 10^−17^) (Figure 3c). Tumors with high CD80 levels exhibited elevated EMT pathway scores, a molecular state critical for the acquisition of invasive properties and dissemination to distant sites.

Furthermore, reinforcing the subtype-specificity observed in BRCA, TIGIT expression in BRCA was found to be inversely correlated with estrogen receptor (ER) signaling activity (FDR = 2.3 × 10^−16^) (Figure S4). This could provide a functional validation for our earlier finding that this checkpoint is enriched in hormone-receptor-negative (basal-like) tumors and could offer a rationale for its poor prognosis in these aggressive subtypes.

### 3.5. Correlation Between CD70, CD80 and TIGIT Expression and Immune Infiltration

We then explored the correlation between mRNA levels and immune infiltration using ImmuCellAI [25]. The immune cell infiltration values estimated by ImmuCellAI reflect relative abundance rather than absolute percentages or infiltration extent in tissue. These are enrichment-based values inferred from gene expression signatures and reflect comparative presence between samples, serving as a proxy for the immune microenvironment.

**Figure 4:**
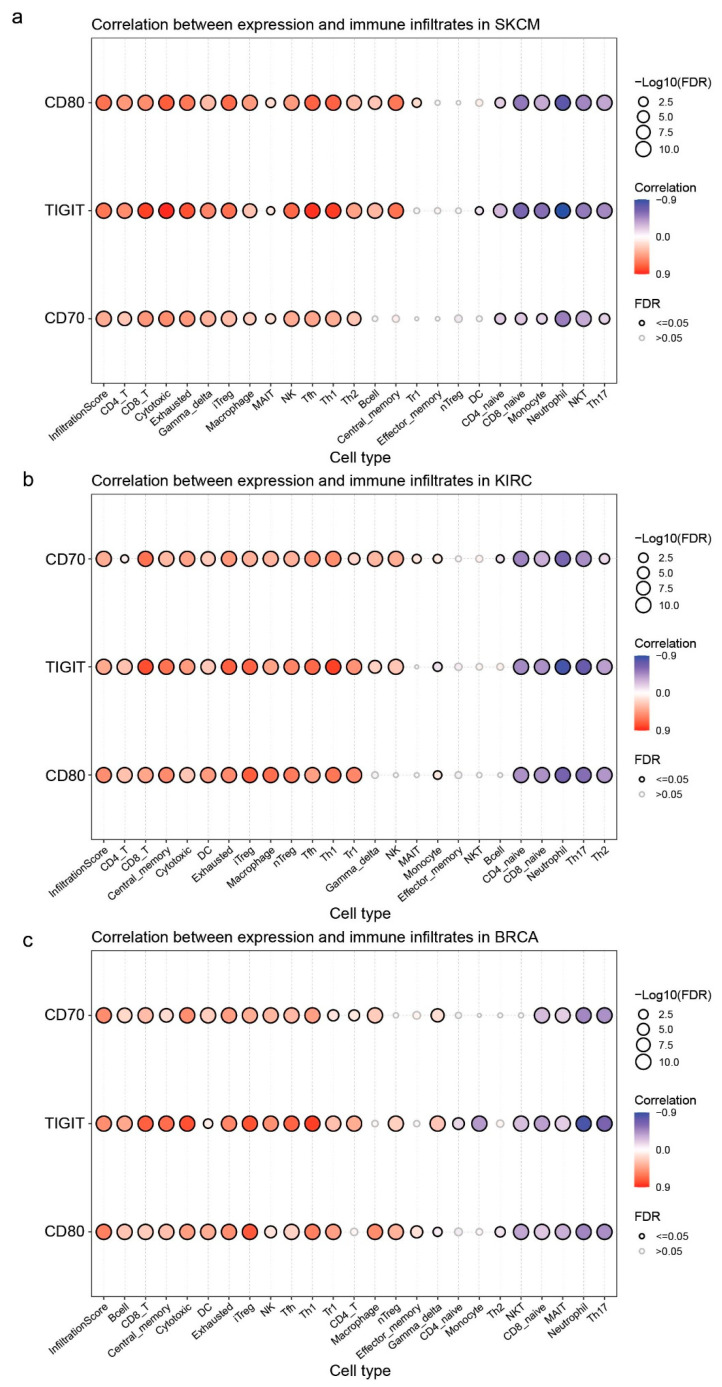
Immune cell infiltration patterns associated with CD70, CD80, and TIGIT. (**a**–**c**) Heatmaps displaying Spearman correlation coefficients between mRNA expression of CD70, CD80, and TIGIT and immune cell infiltration scores estimated by ImmuCellAI in SKCM (**a**), BRCA (**b**), and KIRC (**c**).

In SKCM, BRCA, and KIRC, TIGIT showed the strongest associations with immune infiltration patterns among the three targets (Figure 4a–c). While CD70 and CD80 showed variable association patterns, TIGIT expression consistently displayed a dichotomous relationship with the immune microenvironment, characterized by strong positive correlations with adaptive lineages and deep negative correlations with innate myeloid cells.

**Figure 5:**
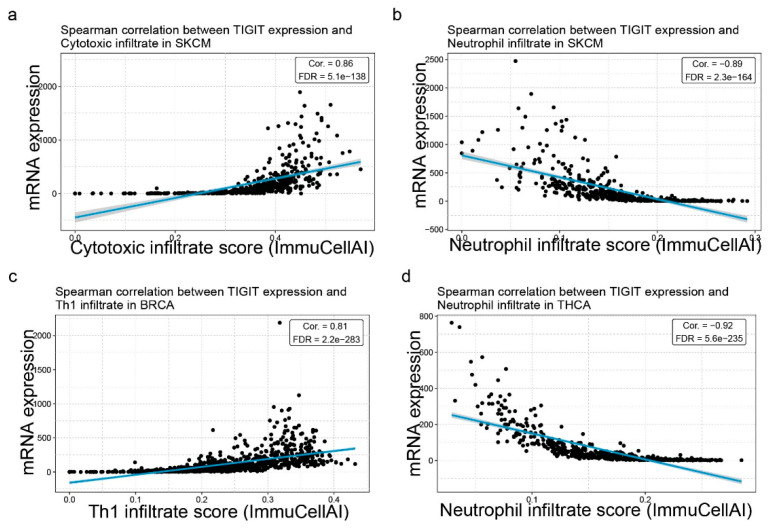
TIGIT expression correlates with cytotoxic immune infiltration and neutrophil exclusion. (**a**) Scatter plot showing the strong positive correlation between TIGIT expression and cytotoxic cell infiltration in SKCM (Cor = 0.86). (**b**) Positive correlation between TIGIT expression and T-helper 1 (Th1) cell abundance in BRCA (Cor = 0.81). (**c**,**d**) Inverse correlations between TIGIT expression and neutrophil infiltration in SKCM (**c**) (Cor = −0.89) and THCA (**d**) (Cor = −0.92).

We identified that TIGIT expression may serve as a surrogate marker for an inflamed, T-cell-rich microenvironment. In SKCM, TIGIT expression was strongly associated with cytotoxic cell infiltration (Cor = 0.86), suggesting that TIGIT is upregulated as a feedback mechanism in tumors under active immune attack (Figure 5a). Similarly, in BRCA, high TIGIT levels correlated significantly with Th1 cell abundance (Cor = 0.81), supporting its association with type 1 immunity, which is generally considered anti-tumor (Figure 5b). We verified this association in KIRC, where TIGIT also showed a robust correlation with T-helper 1 cells infiltration (Cor = 0.81) (Figure S5a).

Conversely, our analysis revealed a strong inverse association with neutrophil infiltration in high-TIGIT tumors. In SKCM, TIGIT was inversely correlated with neutrophil abundance (Cor = −0.89) (Figure 5c). This exclusion was even more pronounced in THCA, where TIGIT expression and neutrophil infiltration displayed a near-perfect negative correlation (Cor = −0.92) (Figure 5d). To validate the universality of this phenomenon, we analyzed the KIRC cohort, which confirmed the previous correlation (Cor = −0.83) (Figure S5b). These data suggest that TIGIT-dominant tumors maintain an immune phenotype that may be associated with reduced presence of granulocytic myeloid cells, highlighting a potential mechanism of immune evasion distinct from CD80/CD70 pathways.

### 3.6. Impact of CD80 DNA Methylation on Immune Cell Infiltration

To elucidate the regulatory mechanisms driving the observed immune landscapes, we profiled the DNA methylation status of the target triad. Among them, only CD80 exhibited a robust, methylation-dependent regulation of immune phenotypes, suggesting a potential role as an epigenetic regulator within the axis.

**Figure 6:**
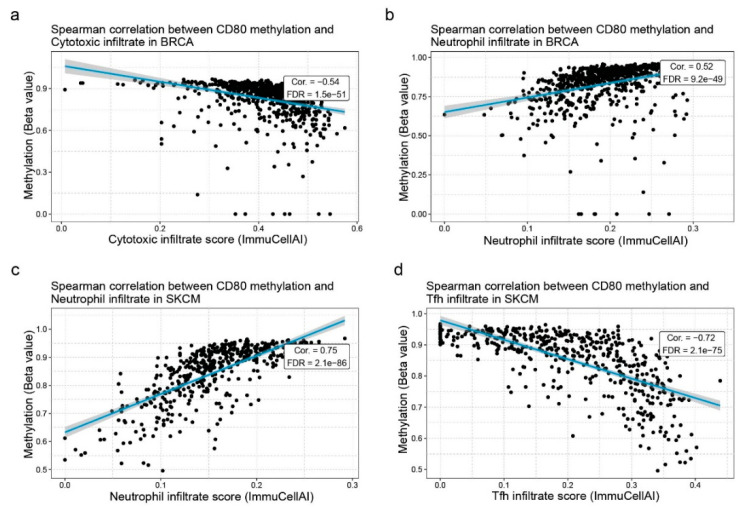
CD80 methylation status influences immune cell infiltration patterns. (**a**,**b**) Scatter plots illustrating the correlation between CD80 promoter methylation and immune cell infiltration in BRCA. (**a**) Negative correlation with cytotoxic T cell infiltrates (Cor = −0.54). (**b**) Positive correlation with neutrophil infiltration (Cor = 0.52). (**c**,**d**) Corresponding methylation-immune cell correlations in SKCM, showing associations with neutrophil (**c**) and T follicular helper (Tfh) cell (**d**) infiltration.

Since promoter methylation typically results in transcriptional silencing, we hypothesized that the epigenetic status of CD80 would mirror the inverse of its expression patterns. Consistent with this hypothesis, hypomethylation of CD80 was strongly associated with immune infiltration patterns indicative of an innate, immune-suppressed microenvironment. In BRCA, high CD80 methylation correlated significantly with the exclusion of cytotoxic infiltrated cells (Cor = −0.54, FDR = 1.5 × 10^−51^), while simultaneously favouring the infiltration of neutrophils (Cor = 0.52, FDR = 9.2 × 10^−49^) (Figure 6a,b). In other tumors we noticed the reverse pattern; for example, in skin melanoma, CD80 methylation was correlated with neutrophil infiltrate and anti-correlated with Tfh infiltrate (Figure 6c,d).

### 3.7. Somatic Mutations in CD80 and TIGIT Drive Lineage-Specific Immune Deviations

Beyond epigenetic regulation, we examined whether somatic mutations in these checkpoints may be associated with altered immune cell recruitment patterns. We identified distinct “mutational immune signatures” associated with CD80 and TIGIT variants.

**Figure 7:**
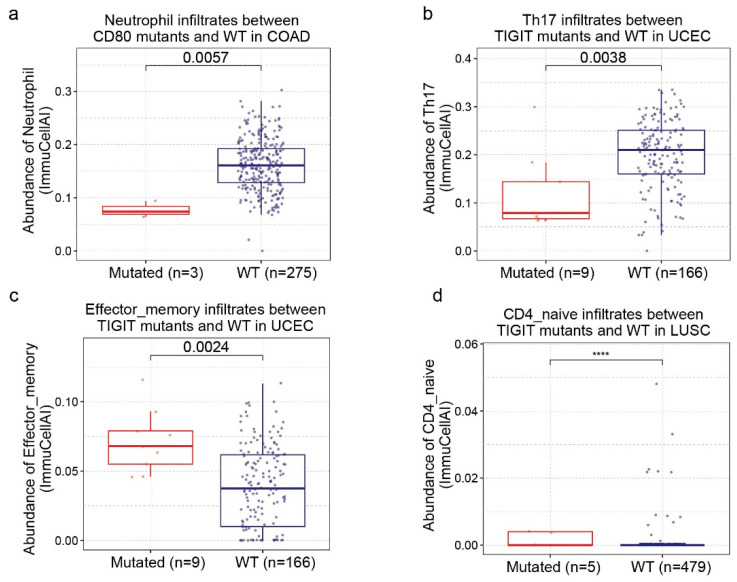
Somatic mutations in CD80 and TIGIT alter the immune microenvironment. (**a**) Box plot comparing neutrophil infiltration between CD80 wild-type (WT) and mutant tumors in colon adenocarcinoma (COAD). (**b**,**c**) Comparative analysis of effector memory T cell (**b**) and Th17 cell (**c**) infiltration between TIGIT WT and mutant tumors in Uterine Corpus Endometrial Carcinoma (UCEC). (**d**) Naïve CD4+ T cell abundance in TIGIT WT versus mutant tumors in Lung Squamous Cell Carcinoma (LUSC). ****, *p* < 0.0001.

In COAD, tumors harboring CD80 mutations exhibited significantly reduced levels of innate effector cell infiltration. Compared to wild-type (WT) tumors, CD80-mutants exhibited significantly reduced infiltration of neutrophils (*p* = 0.0057) (Figure 7a). In contrast, TIGIT mutants were associated with a skewed adaptive T-cell differentiation. In uterine corpus endometrial carcinoma (UCEC), TIGIT showed an abundance in effector memory T cells (*p* = 0.0024) but a significant (*p* = 0.0038) shortage in Th17 cells (Figure 7b,c), suggesting a potential alteration in T-cell differentiation dynamics. Furthermore, in LUSC, TIGIT mutants were linked to an aberrant accumulation of naïve CD4+ T cells compared to the WT cohort (*p* < 0.0001) (Figure 7d).

### 3.8. Genomic Landscape: Mutational Burden and Copy Number Alterations

**Figure 8:**
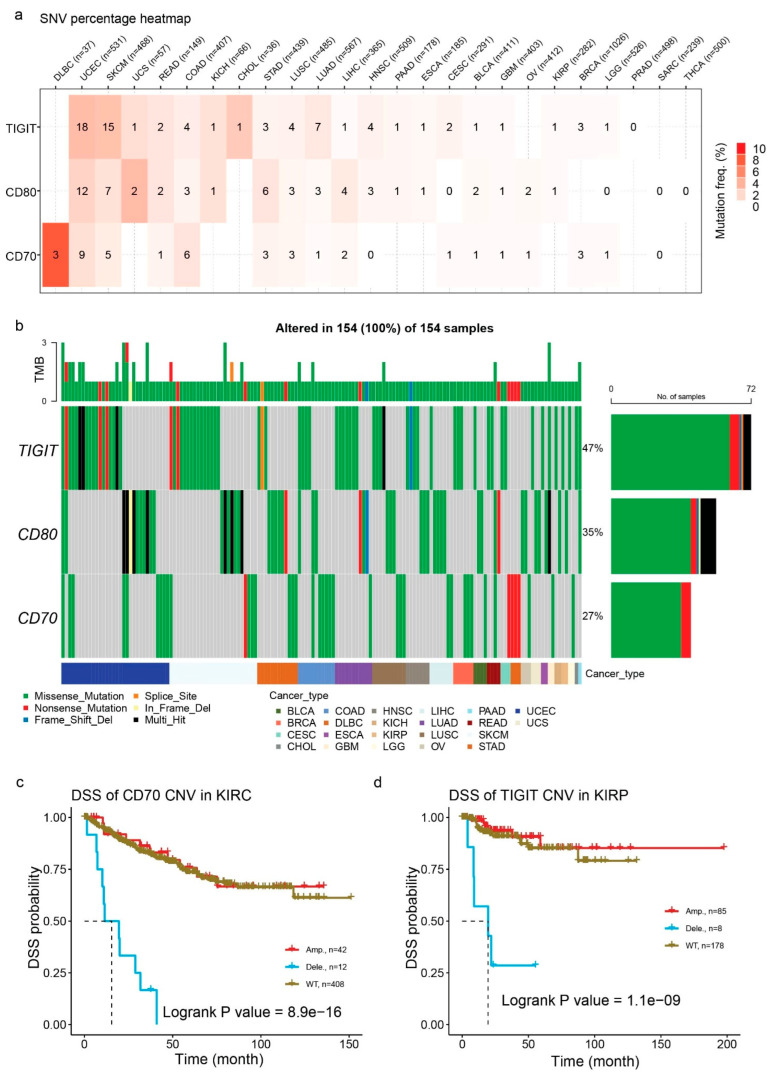
Genomic landscape and prognostic impact of mutations and copy number variations (CNVs). (**a**) Bar chart depicting the frequency of somatic mutations in TIGIT, CD80, and CD70 across cancer types. SKCM and UCEC exhibit the highest mutation rates. (**b**) Oncoplot summarizing the distribution and types of genetic alterations (missense mutations, nonsense mutations, frameshift Indels) in the CD70-CD80-TIGIT axis across pan-cancer cohorts. (**c**) The Kaplan–Meier curves show the DSS in KIRC stratified by CD70 copy number status, demonstrating inferior survival in patients with CD70 amplification. (**d**) Overall Survival analysis in KIRP stratified by TIGIT copy number variation status.

The genomic architecture of TIGIT, CD80, and CD70 is characterized by marked heterogeneity across cancer types, driven by both somatic mutations and extensive copy number alterations (CNV). TIGIT exhibited the highest mutation frequency among the three genes, particularly in SKCM (18%) and UCEC (15%); whereas, CD80 similarly displayed elevated mutational burden in SKCM (12%) and UCEC (7%), distinguishing these immunogenic tumor types as hotspots for checkpoint instability (Figure 8a). Detailed oncoplot analysis suggests that these alterations are predominantly missense mutations, which constitute the majority of variants across the pan-cancer cohort, suggesting a targeted functional modulation of the protein rather than complete loss of function (Figure 8b). This predominance of missense variants is consistent across the gene set, with a median of one variant per sample, as further detailed in the global variant classification metrics (Figure S6a).

Complementing these single nucleotide variants, the cohorts exhibits widespread CNVs (Figure S6b). Heterozygous amplifications and deletions are frequent events too. Notably, CD80 showed a distinct pattern of such amplification in LUSC and ovarian cancer (OV), while CD70 and TIGIT seem to frequently undergo heterozygous deletions in head and neck squamous cell carcinoma (HNSC) and esophageal carcinoma (ESCA) (Figure S6c).

Although less common, homozygous alterations reveal critical specificity. CD70 exhibited homozygous amplifications in LUSC and KIRC, contrasting with the broader heterozygous patterns seen elsewhere (Figure S6d). These genomic events were associated with patient survival outcomes. In KIRC, CD70 amplification is identified as a significant adverse risk factor. Patients harboring this amplification experienced markedly inferior DSS (*p* = 8.9 × 10^−16^) compared to the WT genotypes (Figure 8c). Similarly, TIGIT CNV status serves as a robust biomarker in kidney renal papillary cell carcinoma (KIRP), where amplifications significantly stratify patient survival (*p* = 1.1 × 10^−9^) (Figure 8d), and in UVM, where TIGIT deletions correlate with altered PFS outcomes (*p* = 7.8 × 10^−8^) (Figure S6e).

### 3.9. Methylation-Driven Transcriptional Silencing and Prognostic Value

**Figure 9:**
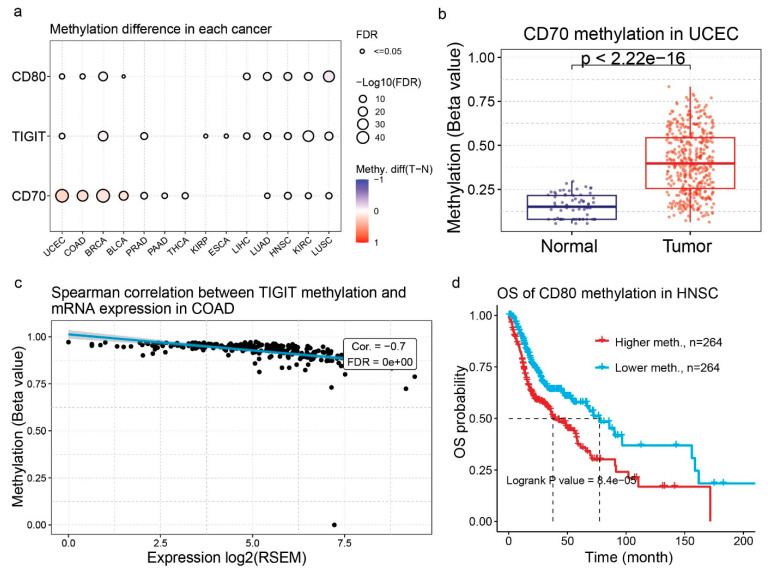
Epigenetic regulation and prognostic significance of DNA methylation. (**a**) Heatmap showing differential promoter methylation of CD70, CD80, and TIGIT across cancer types. Red indicates hypermethylation; blue indicates hypomethylation in tumors. (**b**) Box plot comparing CD70 methylation levels between tumor and normal samples in uterine corpus endometrial carcinoma (UCEC). (**c**) Scatter plot demonstrating the inverse correlation between TIGIT promoter methylation and mRNA expression in colon adenocarcinoma (COAD) (Cor = −0.7). (**d**) The Kaplan–Meier curve shows the OS in head and neck squamous cell carcinoma (HNSC) stratified by CD80 methylation status.

Complementing genomic instability, epigenetic modifications mainly through DNA methylation, have emerged as a dominant mechanism regulating the expression of these immune checkpoints. A systematic analysis of methylation differences revealed a pervasive trend of hypermethylation in tumor tissues, particularly for CD70 and CD80 in LUSC and BRCA (Figure 9a). This dysregulation was most acute in UCEC, where CD70 methylation was significantly higher in tumor samples compared to normals (*p* < 2.2 × 10^−16^), a trend suggesting active epigenetic repression during tumorigenesis (Figure 9b). Such epigenetic remodeling functions as a repressive switch. In COAD, TIGIT promoter methylation was negatively correlated with mRNA expression (Cor = −0.7), confirming that hypermethylation effectively silences gene transcription (Figure 9c). These epigenetic states directly stratify patient outcomes. In HNSC, patients with lower CD80 methylation, indicative of higher gene expression exhibited significantly prolonged OS (*p* = 8.4 × 10^−5^) compared to their hypermethylated counterparts, highlighting the potential of methylation status as a predictive biomarker (Figure 9d).

### 3.10. Pharmacogenomic Profiling Reveals Drug Sensitivity Associations Linked to Checkpoint Expression

To determine the clinical utility of CD70, CD80, and TIGIT beyond immunotherapy, we evaluated the correlation between their mRNA expression and drug sensitivity profiles using large-scale pharmacogenomic datasets. This analysis aimed to explore whether tumors with high checkpoint burden are associated with specific drug sensitivity patterns.

**Figure 10:**
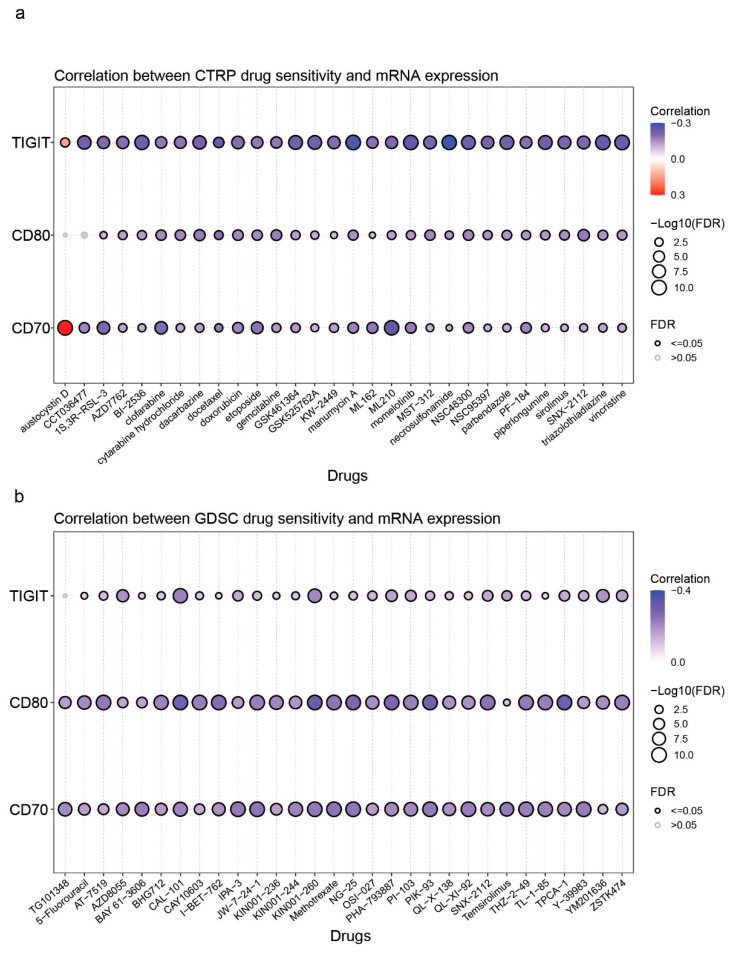
Pharmacogenomic correlations reveal drug sensitivity associations. (**a**) Heatmap displaying Pearson correlation coefficients between CD70, CD80, and TIGIT mRNA expression and drug sensitivity (IC50 values) in the Cancer Therapeutics Response Portal (CTRP) dataset. Negative correlations (blue) indicate increased drug sensitivity with higher gene expression. (**b**) Corresponding pharmacogenomic heatmap using data from the Genomics of Drug Sensitivity in Cancer (GDSC) database. Drug classes include cytoskeletal agents, DNA-damaging compounds, kinase inhibitors, and metabolic pathway modulators.

In CTRP, we observed a distinct pattern where elevated expression of the immune triad was broadly associated with increased drug sensitivity patterns, indicated by negative correlations with IC50 values. CD70 expression displayed a significant correlation with sensitivity to several cytoskeletal and DNA-damaging agents, including vincristine, doxorubicin, and gemcitabine. This suggests that CD70-high tumors, while potentially immune-evasive, may exhibit increased sensitivity in these datasets. Similarly, CD80 expression was associated with sensitivity to the microtubule inhibitor docetaxel and the PLK inhibitor BI-2536, highlighting potential synergy with anti-mitotic therapies. TIGIT showed the broadest set of associations with drug sensitivity, showing strong associations with responsiveness to the topoisomerase inhibitor etoposide and the HSP90 inhibitor SNX-2112 (Figure 10a).

We corroborated these therapeutic associations in the GDSC dataset and uncovered additional targetable pathways. CD70 expression was significantly linked to sensitivity the antimetabolite 5-fluorouracil and the mTOR inhibitor AZD8055. For CD80, we identified a robust association with sensitivity to methotrexate and the PI3K inhibitor PI-103. Consistent with the CTRP findings, TIGIT expression served as a broad predictor of drug responsiveness, particularly towards the mTOR inhibitor temsirolimus and the PAK1 inhibitor IPA-3 (Figure 10b).

Collectively, these pharmacogenomic landscapes indicate that CD70, CD80, and TIGIT expression does not confer broad multi-drug resistance. On the contrary, high expression levels, previously linked to a more differentiated and less stem-like phenotype in our analysis, suggest that a subset of tumors may exhibit specific dependencies on metabolic and cell-cycle pathways that warrant further investigation. Consequently, these molecules may represent candidate biomarkers for predicting therapeutic response, pending further validation.

It is important to note that these pharmacogenomic associations are derived from in vitro cell line models and may not fully recapitulate tumor complexity in vivo. Therefore, these findings should be considered predictive and require further experimental and clinical validation.

### 3.11. Pan-Cancer Associations Between CD70, CD80, TIGIT Expression and Tumor Stemness

**Figure 11:**
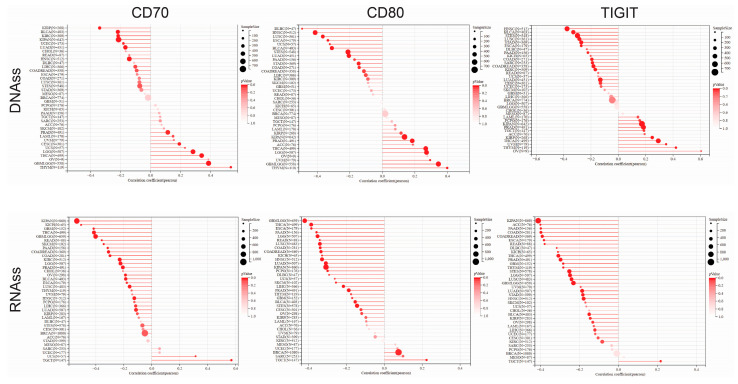
Pan-cancer correlation analysis between CD70, CD80, TIGIT expression and tumor stemness indices. Pan-cancer associations between the expression of CD70, CD80, and TIGIT and tumor stemness were evaluated across TCGA cancer types using the DNA methylation–based stemness score (DNAss) (upper panels) and the RNA expression–based stemness score (RNAss) (lower panels). Gene expression values were log_2_(x + 0.001)–transformed prior to analysis. Pearson’s correlation coefficients were calculated within each cancer type. Tumor types are ordered according to correlation strength. Dot size represents the number of samples per cancer type, while color intensity indicates the magnitude and direction of the correlation coefficient. DNAss, DNA methylation–based stemness score; RNAss, RNA expression–based stemness score.

We also investigated the relationship between the expression of CD70, CD80, and TIGIT and tumor stemness features across multiple cancer types using DNAss and RNAss scores (Figure 11).

Across the pan-cancer cohort, CD70 expression was predominantly negatively correlated with DNAss in most tumor types, suggesting that higher CD70 expression is generally associated with reduced DNA methylation–based stemness. However, the strength of this association varied substantially among cancers, indicating pronounced tumor-specific heterogeneity.

Similarly, CD80 expression showed largely negative correlations with DNAss across the majority of cancer types, although the magnitude of these correlations differed between tumors. Only a limited number of cancers displayed weak or positive associations, highlighting context-dependent relationships between CD80 expression and epigenetic stemness.

For TIGIT, DNAss correlations were also predominantly negative across cancer types, with several tumors exhibiting relatively strong inverse associations, while others showed weaker or near-neutral correlations.

Analysis of RNAss revealed a broadly consistent pattern. Expression of CD70, CD80, and TIGIT was generally negatively correlated with transcriptional stemness across most tumor types. Compared with DNAss, RNAss correlations appeared more uniform in direction, although their magnitude varied across cancers. Notably, CD70 and TIGIT demonstrated particularly pronounced inverse correlations with RNAss in multiple tumor types, whereas CD80 showed more moderate but still predominantly negative associations.

Collectively, these pan-cancer analyses indicate that increased expression of CD70, CD80, and TIGIT is generally associated with lower stemness features at both the epigenetic and transcriptional levels, while underscoring substantial inter-tumoral heterogeneity in the strength of these relationships.

### 3.12. Single-Cell Transcriptomic Profiling of CD70, CD80, and TIGIT in Select Tumor Microenvironments

Single-cell RNA sequencing (scRNA-seq) analysis from the TISCH2 database revealed distinct expression patterns of the immunoregulatory molecules CD70, CD80, and TIGIT across different cellular populations of the TME in selective cancer samples. We prioritized CD80 in HNSC, where our data previously established methylation as a key prognostic switch. scRNA-seq analysis resolved this by demonstrating a strictly lineage-restrictive expression pattern enriched in monocytes and macrophages (logTPM = 1.13) (Figure S7a), rather than malignant cell, supporting its proposed role as a protective, co-stimulatory signal within the myeloid compartment.

For TIGIT, we focused on SKCM, identified as the distinct hotspot for TIGIT-mediated immune modulation and high mutational burden. Single-cell resolution confirmed its confinement to exhausted T cells and NK cells (Figure S7b), supporting its potential utility as a surrogate marker for immunogenicity and a prime target for reversing exhaustion in “hot” tumor microenvironments.

Finally, CD70 was characterized in LUSC to investigate the paradox of simultaneous homozygous amplifications and promoter hypermethylation, allowing us to delineate whether the gene is epigenetically silenced or aberrantly expressed on malignant populations. Our analysis suggests that genomic amplification overrides epigenetic silencing, associated with increased CD70 expression specifically on malignant tumor cells, thereby highlighting it as a direct, tumor-intrinsic target for antibody-drug conjugates in this histological subtype (Figure S7c).

## 4. Discussion

Cancer progression is driven not only by intrinsic genetic alterations but also by dynamic interactions with the tumor microenvironment (TME), particularly immune evasion mechanisms [27,28,29]. Immune checkpoint molecules play a central role in regulating the balance between anti-tumor immunity and immune suppression. Despite the clinical success of immune checkpoint inhibitors, therapeutic responses remain limited to a subset of patients, largely due to tumor heterogeneity, immune suppression, and impaired antigen presentation [30,31,32,33,34]. In this study, we performed an integrative pan-cancer analysis of CD70, CD80, and TIGIT, identifying their coordinated dysregulation across malignancies and highlighting their potential as composite biomarkers of tumor–immune interactions. Our findings align with emerging evidence emphasizing the importance of multi-omics and single-cell approaches in understanding immune regulation and tumor heterogeneity [35].

Importantly, while the integrative multi-omics framework employed in this study enables robust cross-validation across independent datasets and analytical layers, these findings remain inherently computational and hypothesis-generating. As such, the biological and clinical relevance of the identified associations requires independent validation using experimental systems and prospective clinical cohorts. In particular, functional assays will be necessary to determine whether the observed relationships reflect causal mechanisms or indirect associations driven by the tumor microenvironment.

### 4.1. Clinical Relevance and Prognostic Associations

We observed marked dysregulation of CD70, CD80, and TIGIT across multiple cancer types, with particularly strong upregulation in renal malignancies. These findings are consistent with prior studies demonstrating the involvement of TIGIT in renal carcinoma immune suppression and its association with adverse outcomes [36,37,38]. Similarly, CD70 overexpression has been linked to tumor progression and poor prognosis across multiple malignancies, including renal and colorectal cancers [7,38,39]. Survival analyses further identified tumor-specific prognostic associations, most notably the strong link between high TIGIT expression and poor outcomes in uveal melanoma (UVM). These results are consistent with previous pan-cancer studies and epigenetic analyses demonstrating that TIGIT expression and methylation status are associated with survival and immune infiltration in UVM [40,41,42,43,44]. In parallel, CD70 expression was associated with shorter disease-free intervals in renal cancer, supporting its role as a clinically relevant biomarker [45].

However, it is important to emphasise that these survival analyses are based on univariate approaches and should be interpreted as correlative rather than indicative of independent prognostic value, as multivariate modelling was not feasible due to incomplete clinical annotation [45].

### 4.2. Checkpoint Expression and Oncogenic Signalling

Beyond clinical associations, our results suggest that CD70, CD80, and TIGIT are linked to key oncogenic pathways. In particular, we identified associations between checkpoint expression and epithelial–mesenchymal transition (EMT), apoptosis, and hormone receptor signalling. For example, CD80 expression was associated with EMT pathway activity in bladder cancer, consistent with the established role of EMT in tumor progression and metastasis [46,47]. This observation is supported by studies demonstrating that EMT is closely linked to immune evasion and checkpoint regulation across cancer types [46,48]. In breast cancer, TIGIT expression was associated with apoptosis-related signatures and inversely correlated with estrogen receptor signalling. These findings are consistent with previous reports linking TIGIT expression to immune infiltration and aggressive tumor subtypes, particularly in hormone receptor–negative breast cancers [11,49,50,51]. Importantly, these associations likely reflect the presence of an inflamed TME rather than direct mechanistic effects of checkpoint molecules.

### 4.3. Immune Microenvironment and Checkpoint Regulation

Analysis of immune infiltration patterns revealed that TIGIT is strongly associated with a T-cell–inflamed microenvironment, characterised by enrichment of cytotoxic and Th1 cell populations. This is consistent with its established role as a marker of activated yet functionally exhausted T cells in multiple cancer types, including melanoma, breast cancer, and renal carcinoma [52,53,54,55]. Conversely, we observed a consistent inverse association between TIGIT expression and neutrophil infiltration. This finding may reflect distinct immune microenvironmental states, as tumor-associated neutrophils have been linked to poor prognosis and tumor progression in several cancers, including renal carcinoma [56]. Together, these observations support the concept that TIGIT marks tumors undergoing active immune engagement but characterized by functional exhaustion.

### 4.4. Genomic and Epigenetic Determinants

Our multi-omics analysis indicates that checkpoint dysregulation is shaped by a complex interplay of genomic and epigenetic mechanisms. High mutation frequencies in SKCM and UCEC are consistent with the known mutational burden of these tumor types [57,58], while somatic mutations in immune-related genes have been shown to influence tumor behavior and immune composition [59]. We further demonstrate that copy number alterations, particularly CD70 amplification in renal cancer, are associated with adverse clinical outcomes, in agreement with previous reports linking CD70 expression to poor prognosis [45,60]. In parallel, DNA methylation emerged as a key regulatory mechanism, particularly for CD80 and TIGIT. Hypermethylation was associated with transcriptional repression, consistent with established epigenetic regulation of immune checkpoint genes [3,61,62].

Interestingly, the coexistence of CD70 amplification and promoter hypermethylation in LUSC highlights the complexity of gene regulation, suggesting that genomic alterations may override epigenetic repression in certain contexts [3]. These findings are in line with previous studies demonstrating that methylation patterns can define tumor immunogenicity and clinical outcomes across cancers [63,64].

### 4.5. Pharmacogenomic Associations and Therapeutic Implications

Our pharmacogenomic analyses identified associations between checkpoint expression and sensitivity to a broad range of anticancer agents. These findings suggest that tumors with high checkpoint expression may exhibit distinct therapeutic vulnerabilities, supporting the potential for combinatorial strategies integrating immunotherapy with conventional treatments.

However, these associations are derived from large-scale cell line datasets and should be interpreted as predictive and hypothesis-generating. As highlighted in previous studies, pharmacogenomic correlations provide valuable insights into drug response mechanisms but require validation in clinical settings [65,66,67,68,69]. Accordingly, further experimental and translational studies are necessary to determine the clinical relevance of these observations.

### 4.6. Single-Cell Resolution of Checkpoint Expression

Single-cell transcriptomic analyses provided important insights into the cellular context of checkpoint expression. CD80 was predominantly expressed in myeloid populations, consistent with its role in antigen-presenting cells, whereas TIGIT expression was confined to exhausted T cells and NK cells, in agreement with previous studies [52,65,66,67,70]. In contrast, CD70 expression in certain cancers was observed within malignant cells, supporting its role as a tumor-intrinsic immunomodulatory factor [68,69].

These findings highlight the importance of cellular context in interpreting immune checkpoint biology and underscore the limitations of bulk transcriptomic analyses.

### 4.7. Limitations and Future Perspectives

Several limitations should be acknowledged. First, all analyses are based on publicly available datasets and are therefore inherently correlative, precluding direct inference of causal relationships. Although the use of large-scale, multi-cohort datasets enhances statistical robustness, it does not substitute for experimental validation. Second, immune infiltration estimates rely on computational methods that may be influenced by tumor purity and dataset variability [25]. Third, survival analyses were primarily conducted using univariate approaches due to incomplete clinical annotation across cohorts, limiting the ability to establish the independent prognostic value of the studied biomarkers. Fourth, pharmacogenomic associations were derived from large-scale cancer cell line datasets, which may not fully capture the complexity of TME interactions observed in vivo. Fifth, single-cell transcriptomic analyses were restricted to selected datasets and cancer types and therefore may not fully represent the heterogeneity of checkpoint expression across all malignancies.

Collectively, our findings support the role of CD70, CD80, and TIGIT as integrative biomarkers of tumor progression and immune contexture. However, given the computational and correlative nature of this study, these results should be considered hypothesis-generating. Future studies incorporating experimental validation, spatial profiling, and clinically annotated cohorts will be essential to confirm these associations and facilitate their translation into precision immuno-oncology applications.

## 5. Conclusions

In conclusion, this comprehensive pan-cancer bioinformatic analysis establishes CD70, CD80 and TIGIT as a multifaceted biomarker signature associated with tumor progression, immune contexture, and therapeutic response. While these findings provide a robust framework for understanding their roles in cancer biology, they remain hypothesis-generating and require further validation. Nevertheless, this study highlights the value of multi-omics integration in identifying clinically relevant immune biomarkers and informing precision immuno-oncology strategies.

## Data Availability

Genomic data were extracted from TCGA (https://portal.gdc.cancer.gov/, accessed on 1 February 2025) and GTEx (https://www.gtexportal.org/home/, accessed on 1 February 2025).

## Supplementary Materials

The following supporting information can be downloaded at https://www.mdpi.com/article/10.3390/cimb48060641/s1.
