# Supplementary material for: Comprehensive Bioinformatic Characterization of CD70, CD80, and TIGIT as Diagnostic, Prognostic, and Immune Biomarkers in Pan-Cancer
Source: Curr Issues Mol Biol. 2026 Jun 21;48(6):641. doi: 10.3390/cimb48060641 (PMC13297921; doi:10.3390/cimb48060641)
Supplement: Supplementary file 1 [file cimb-48-00641-s001.zip › cimb-4319209-Supplementary material.pdf]

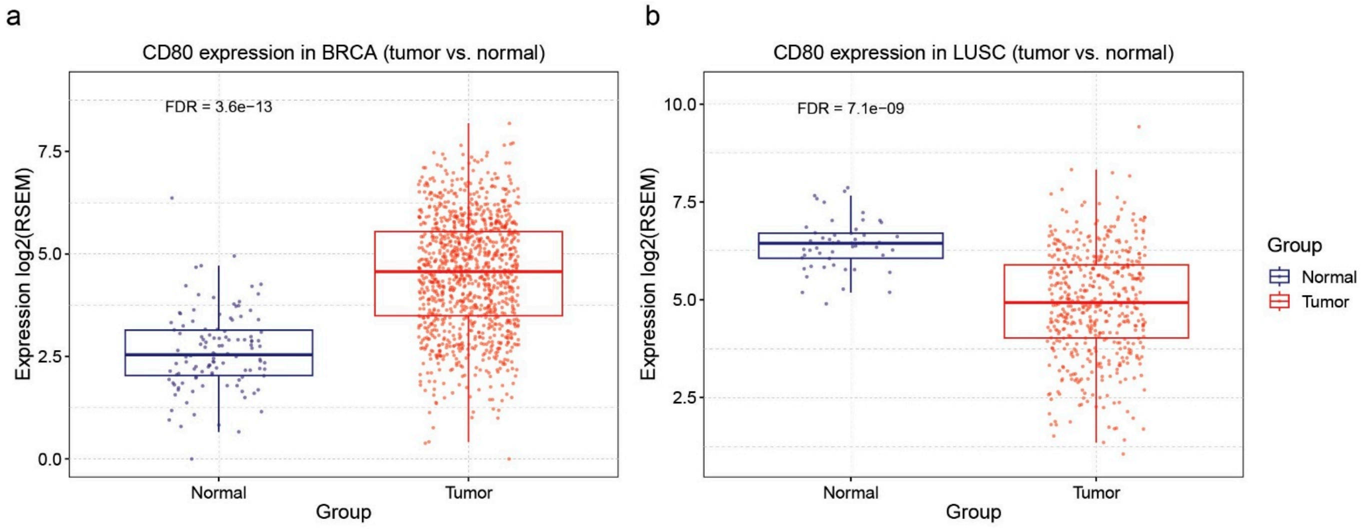

Figure S1. Differential expression of CD80 in breast cancer (BRCA) and lung squamous cell carcinoma (LUSC). (a) Box plot showing CD80 mRNA overexpression in BRCA tumor samples compared with normal breast tissues (FDR = 3.6×10^{-13}). (b) Box plot depicting significantly lower CD80 expression in LUSC relative to matched normal lung tissues (FDR = 7.1×10^{-9}). Expression values based on $\log_2$(RSEM) normalization from TCGA and GTEx datasets.

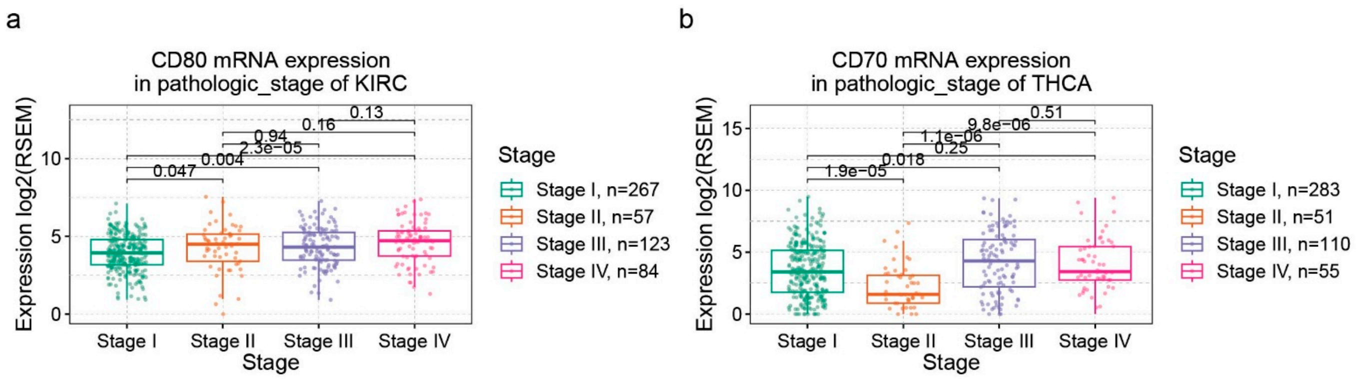

Figure S2. Stage-dependent expression patterns of CD70 and CD80. (a) CD80 expression across pathological stages in KIRC. (b) CD70 overexpression in late-stage thyroid carcinoma (THCA), with significantly higher levels in stage III/IV tumors. All statistical comparisons performed using FDR-corrected ANOVA tests.

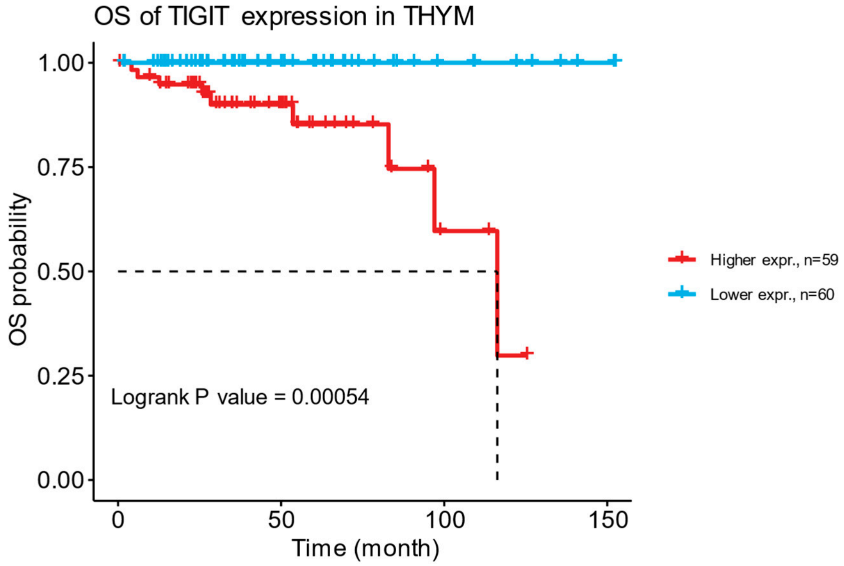

Figure S3. Prognostic relevance of TIGIT expression in thymoma (THYM). Kaplan–Meier overall survival curve comparing high- versus low-TIGIT expression groups in THYM patients. Elevated TIGIT levels are associated with significantly reduced overall survival (OS; log-rank p = 0.00054). Patients were stratified by median TIGIT expression.

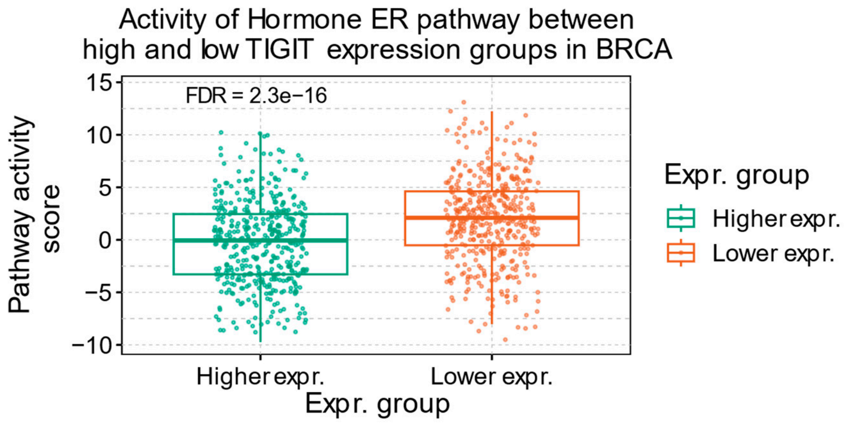

Figure S4. Negative correlation between TIGIT expression and estrogen receptor (ER) signaling in breast cancer (BRCA). Scatter plot showing a strong inverse association between TIGIT expression and ER pathway activity in BRCA (FDR = 2.3×10^{-16}), indicating preferential enrichment of TIGIT in ER-negative/basal-like tumors.

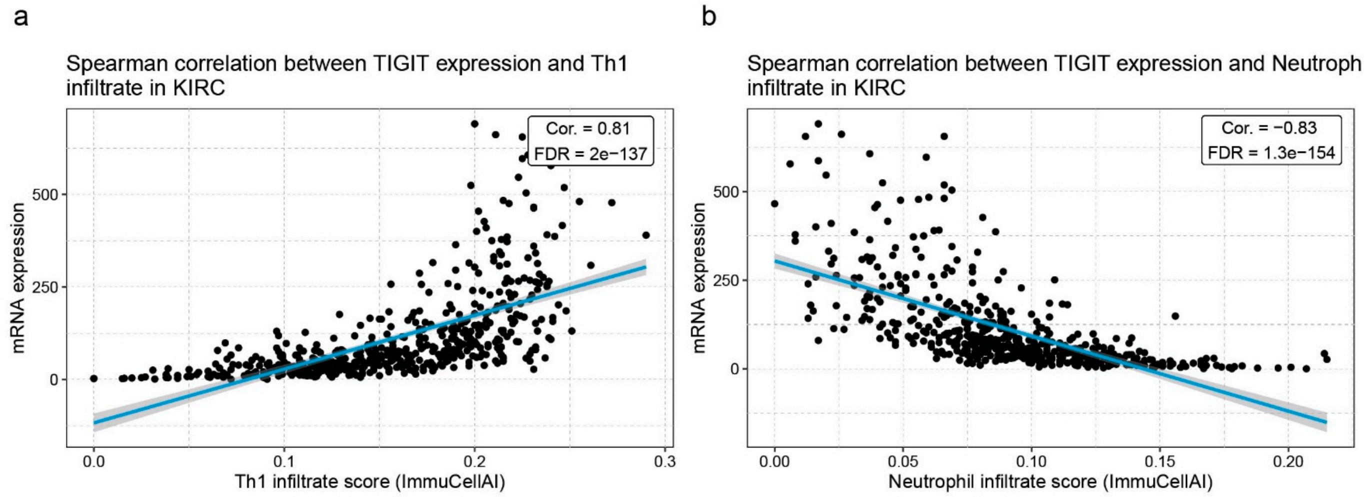

Figure S5. TIGIT-associated immune infiltration patterns in KIRC. (a) Positive correlation between TIGIT expression and T-helper 1 (Th1) cell infiltration (Cor = 0.81). (b) Strong negative correlation between TIGIT expression and neutrophil infiltration (Cor = −0.83). Immune cell scores were computed using ImmuCellAI. Statistical significance based on Spearman correlation.

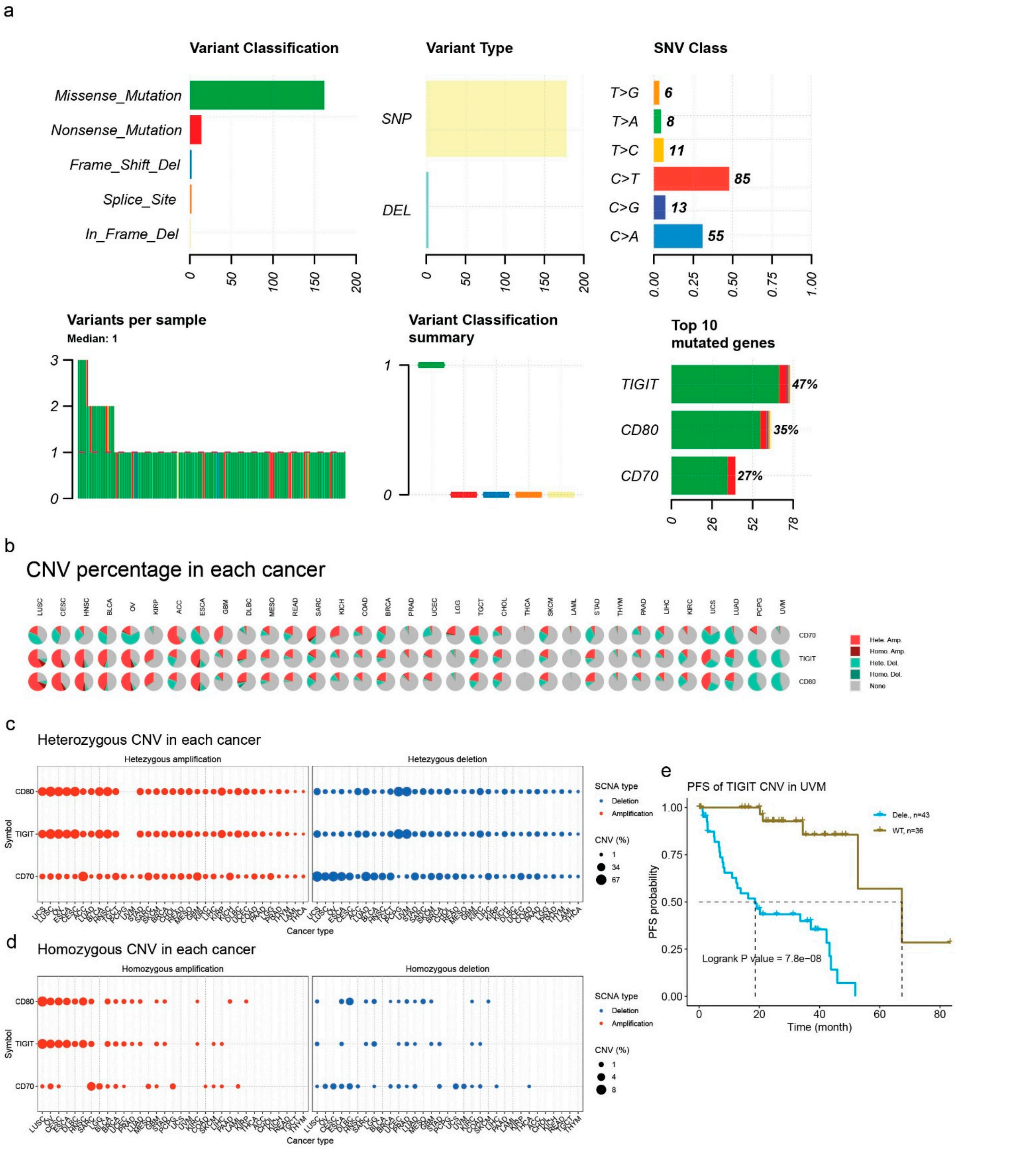

Figure S6. Genomic and CNV landscape of CD70, CD80, and TIGIT across cancers. (a) Summary of variant classification across the CD70–CD80–TIGIT axis, showing predominance of protein-altering missense mutations. (b) Frequency of copy number alterations (CNVs) across tumor types. (c) Heterozygous amplifications and deletions of CD70, CD80 and TIGIT in each cancer. (d) Homozygous amplifications and deletions of CD70, CD80 and TIGIT in each cancer. (e) Progression-frere survival (PFS) of TIGIT CNV in uveal melanoma (UVM). Wild-type (WT) patients exhibited significantly better PFS survival. Data derived from >10,000 TCGA samples.

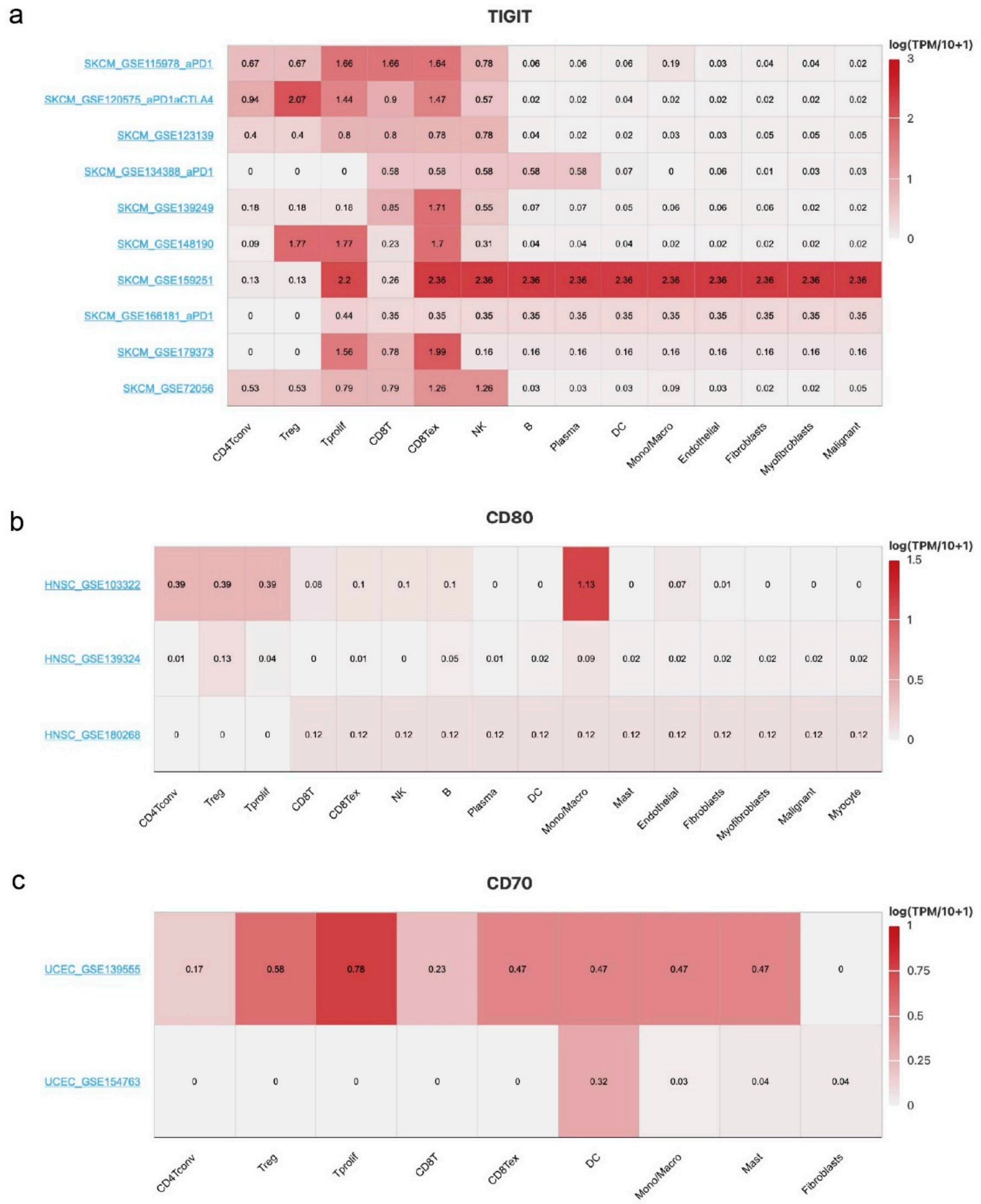

Figure S7. Single-cell transcriptomic localization of CD80, TIGIT, and CD70 across tumor microenvironments. (a) TIGIT expression in various skin cutaneous melanoma (SKCM) datasets, was enriched in exhausted T cells and NK cells. (b) CD80 expression in various head and neck squamous cell carcinoma (HNSC) datasets, was predominantly restricted to monocytes and macrophages (logTPM = 1.13). (c) CD70 expression in two lung squamous cell carcinoma (LUSC) datasets, driven by genomic amplification and localized primarily to malignant epithelial cells. Single-cell profiles were obtained from TISCH2 datasets.
